# Plasmatic Levels of HSP90α at Diagnosis: A Novel Prognostic Indicator of Clinical Outcome in Advanced Lung Cancer Patients Treated With PD-1/PD-L1 Inhibitors Plus Chemotherapy

**DOI:** 10.3389/fonc.2021.765115

**Published:** 2021-12-03

**Authors:** Shubin Chen, Qitao Yu, Shaozhang Zhou

**Affiliations:** Medical Oncology of Respiratory, Guangxi Cancer Hospital and Guangxi Medical University Affiliated Cancer Hospital, Nanning, China

**Keywords:** advanced lung cancer, HSP90α, PD-1/PD-L1 inhibitors, chemotherapy, prognostic marker

## Abstract

**Background:**

The purpose of this study was set to investigate the prognostic role of plasmatic levels of heat shock protein 90 alpha (HSP90α) at diagnosis in advanced lung cancer patients treated with Programmed cell death protein 1 (PD-1)/Programmed cell death-Ligand protein 1 (PD-L1) inhibitors plus chemotherapy.

**Methods:**

A total of 137 advanced lung cancer patients treated with PD-1/PD-L1 inhibitors plus chemotherapy admitted to the Guangxi Medical University Cancer Hospital were enrolled in this study. Smooth curve fitting was conducted to address the nonlinearity of HSP90α and progression-free survival (PFS) and overall survival (OS). We calculated the inflection point using a recursive algorithm. Kaplan–Meier survival analysis and Cox proportional hazards regression model were used to assess the prognostic value of HSP90α for PFS and OS. Subgroup analysis was performed to evaluate the relationship between high HSP90α and disease progression and death risk.

**Results:**

The average age of patients was 58.6 ± 9.8 years, and 73.7% of them were men. We divided patients according to their plasmatic levels of HSP90α into low (HSP90α <52.7 ng/ml) group and high (HSP90α ≥52.7 ng/ml) group. Kaplan–Meier analysis showed a shorter PFS and OS for the high group with log-rank P < 0.05. Univariate and multivariate analyses indicated that high HSP90α was associated with an increased risk of disease progression and death after fully adjusting potential confounders with hazard ratio (HR) 1.8 (95% CI = 1.0–3.2) and HR 2.4 (95% CI = 1.1–5.1), respectively (P < 0.05). After stratification by subgroup analysis, the relationship between high HSP90α and the risk of disease progression and death was consistent across all patient subgroups.

**Conclusion:**

Plasmatic levels of HSP90α at diagnosis can be considered a potential independent prognostic marker of advanced lung cancer patients treated with PD-1/PD-L1 inhibitors plus chemotherapy. A further large-scale prospective validation study is needed to determine whether these results are widely applicable.

## Introduction

Lung cancer is the leading cause of cancer-associated mortality all over the world (1), including non-small cell lung cancer (NSCLC) and small cell lung cancer (SCLC). Despite advances in lung cancer diagnostics and therapeutics, the survival rate of patients with advanced lung cancer, calculated based on 5 years, remains poor (2).

The standard systemic therapeutic plans for advanced lung cancer patients have made significant progress in recent years, such as chemotherapy, targeted therapy, and immune checkpoint inhibitors (ICIs). The combination of immunotherapy with a plethora of different agents (chemotherapy, antiangiogenic, and other immunotherapeutic drugs) was supported with ample evidence rationale. PD-1 and its ligand (PD-L1) extensively entered clinical practice for the management of advanced lung cancer patients. An overview of current clinical experimental results, including KEYNOTE-189, KEYNOTE-407, IMPOWER133, CASPIAN, and others (3–6), demonstrated that PD-1/PD-L1 inhibitors plus chemotherapy are gradually emerging as a novel therapeutic paradigm for advanced lung cancer patients. Currently, biomarkers that could discriminate patients who benefit from immunotherapies are still under investigation. Therefore, exploration of indicators to predict prognosis and immunotherapy response is urgently warranted, which would help clinicians to optimize subsequent treatment strategies.

Heat shock protein (HSP)27, HSP40, HSP60, HSP70, HSP90, and HSP110 are six prominent families of HSPs (7). HSP90 has two isoforms: HSP90α and HSP90β, the levels of plasma HSP90α are extremely low in normal. However, in many diseased states such as neoplasms, HSP90α can be secreted explicitly to the extracellular space, entering the blood circulation (8). A previous study indicated that plasmatic levels of HSP90α are significantly elevated in patients with lung cancer compared with those of healthy controls (9). Meanwhile, a multitude of studies have demonstrated that the HSP90α was associated with tumor cell migration, invasion, and metastasis (10–12).

However, whether plasmatic levels of HSP90α at diagnosis could predict PD-1/PD-L1 inhibitors plus chemotherapy efficacy remains unknown. Thus, this retrospective cohort study was conducted to assess the prognostic significance of plasmatic levels of HSP90α at diagnosis in advanced lung cancer patients treated with PD-1/PD-L1 inhibitors plus chemotherapy.

## Materials and Methods

### Study Design and Participants

**Figure 1:**
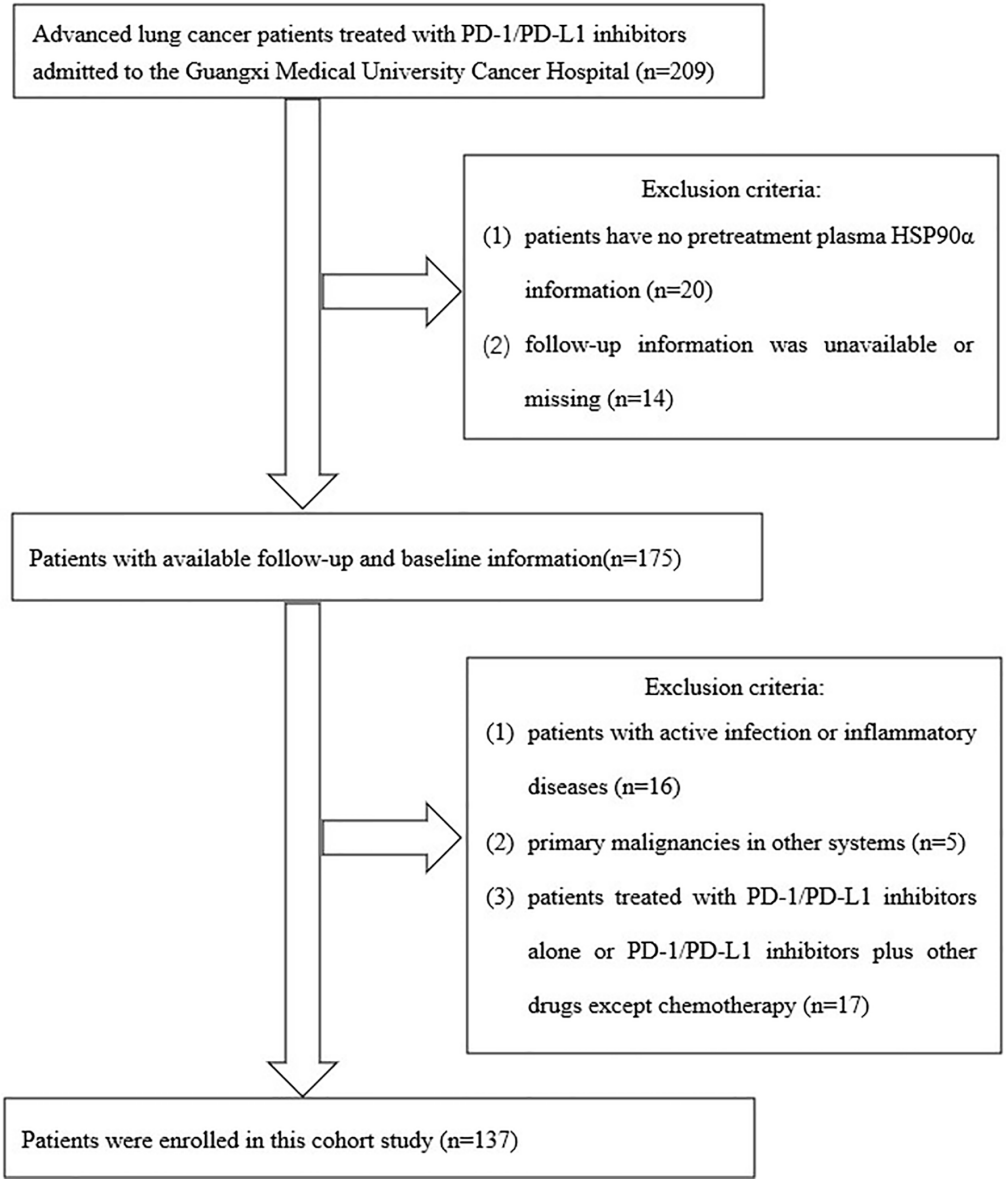
Flowchart of the study.

A total of 205 advanced lung cancer patients treated with PD-1/PD-L1 inhibitors admitted to the Guangxi Medical University Cancer Hospital were collected in this study. The inclusion criteria of this study were the following: (1) pathologically confirmed primary lung cancer according to the third-version 2015 WHO histological classification of lung cancer; (2) clinical stage IIIB/IIIC or IV according to the eighth TNM staging system of lung cancer; (3) Eastern Cooperative Oncology Group (ECOG) score of 0–3 points for the physical status; (4) patients treated with PD-1/PD-L1 inhibitors. Exclusion criteria were as follows: (1) Follow-up information was unavailable and the cases with missing data; (2) patients with active infection or inflammatory diseases that may potentially interfere with the outcome analysis before blood biochemistry assay; (3) primary malignancies in other systems; (4) patients treated with PD-1/PD-L1 inhibitors alone or PD-1/PD-L1 inhibitors plus other drugs except for chemotherapy. After screening by inclusion and exclusion criteria, a total of 137 patients met the final analysis criteria. The screening process and results are shown in **Figure 1**.

According to National Comprehensive Cancer Network (NCCN) guidelines for patients with lung adenocarcinoma, we routinely perform molecular testing on these patients, including epidermal growth factor receptor (EGFR), anaplastic lymphoma kinase (ALK), ROS proto-oncogene 1 (ROS1), mesenchymal to epithelial transition (MET), RET proto-oncogene (RET), BRAF gene (BRAF), kirsten rat sarcoma viral oncogene (KRAS), human epidermal growth factor receptor-2 (HER-2). According to the results of existing clinical trials, the efficacy of PD-1/PD-L1 inhibitors plus chemotherapy in patients with gene mutations is not apparent, and the corresponding gene mutations can be treated with targeted drugs. Therefore, among the patients enrolled in our experiment, the proportion of patients with mutations, as mentioned above, was relatively small. In addition, the types of mutation patients are not evenly distributed, and this experiment did not group them into groups based on gene mutations.

All selected patients received standard chemotherapy regimens in the combined treatment. For patients with non-small cell lung cancer, according to NCCN guidelines, we chose a platinum-based (cisplatin or carboplatin) chemotherapy regimen combined with pemetrexed, paclitaxel, paclitaxel liposome, albumin paclitaxel, or docetaxel for treatment. All small cell lung cancer patients received chemotherapy that consisted of platinum (cisplatin or carboplatin) plus etoposide combination in 4–6 chemotherapy cycles. In terms of PD-L1/PD-1 inhibitors, PD-L1 inhibitors included atezolizumab and durvalumab. PD-1 inhibitors included pembrolizumab, nivolumab, camrelizumab, tislelizumab, sintilimab, and toripalimab.

The patients were routinely followed up every 3 months. We maintained the follow-up by retrieving follow-up medical records stored in the outpatient database. The patients were followed by personal contact with our professional follow-up institution, which involved requests for information about tumor recurrence and survival status. Tumor lesions before and after PD-1/PD-L1 inhibitors plus chemotherapy were observed and measured by CT examinations to assess efficacy. The efficacy was classified as complete response (CR), partial response (PR), stable disease (SD), and progressive disease (PD) according to the Response Evaluation Criteria In Solid Tumors (RECIST) criteria (version 1.1).

### Plasmatic Levels of HSP90α Analysis

The Quantitative Detection Kit for Heat Shock Protein 90α (ELISA) produced by Yantai Protgen Biotechnology Development Co., Ltd., was used to quantitate plasmatic levels of HSP90α; we strictly followed the manufacturer’s instructions for testing. The processes were as follows: 1) Collect peripheral venous blood from the enrolled patients and store it in EDTA-K2 anticoagulation tubes, centrifuge the blood to obtain a plasma sample. 2) Add the calibrator to 0.4 ml of analyte diluent to dissolve and mix and use the diluent to dilute the sample to be tested 20 times. 3) Set the calibrator hole and sample hole, add 50 μl of the calibrator and diluted sample respectively. 4) Add 50 μl of labeling solution for HSP90α to each hole and shake gently to mix. 5) Cover them with sealing film and incubate at 37°C for 60 min. 6) Remove the reaction solution, add 300 μl of washing solution to each hole, wash the plate six times in total. 7) Add 50 μl of color developer A and B to each hole, shake gently to mix, and incubate at 37°C for 20 min. 8) Add 50 μl of stop solution to each hole to stop the color development. 9) Record the optical density (OD) at 450-nm/620 (630)-nm wavelength within 10 min of adding the stop solution. 10) Use the software that comes with the instrument, take the logarithm of the concentration of the calibrator 1–5 as the X-axis and the logarithm of the calibrator’s OD value as the Y-axis, draw a standard curve, and substitute the logarithm of sample’s OD value into the regression equation to calculate the sample’s HSP90α value.

### Data Collection

Data of all patients pertaining to clinicopathological variables and HSP90α were extracted from medical records, including histological type, sex, age, ECOG score, clinical stage, smoking history, and treatment plan.

Progression-free survival (PFS) was calculated from the start date of treatment to the date of objective disease progression; alternatively, if no disease progression was recorded, to the time of the last follow-up. Overall survival (OS) was defined as the time between treatment to the last follow-up or death date.

This study was approved by the ethics committee of Guangxi Medical University Affiliated Cancer Hospital. All procedures performed in this study involving human participants followed the ethical standards of our institution’s research committee.

### Statistical Analyses

Continuous variables are expressed as mean ± standard deviation (SD) for normally distributed variables. Categorical variables are presented as frequency, percentage, or ratio. We used χ^2^ or Fisher’s exact test (categorical variables), Student’s t-test (normal distribution), or Mann–Whitney U test (skewed distribution) to test differences between HSP90α groups.

Cox proportional hazards regression model with cubic spline functions and smooth curve fitting (penalized spline method) was conducted to address the nonlinearity of pretreatment plasma HSP90α and PFS and OS. We calculated an inflection point of 52.7 ng/ml. The effects of plasmatic levels of HSP90α at diagnosis on PFS and OS were evaluated using Kaplan–Meier curves (log-rank test). Subgroup analysis was used to elaborate the relationship between high HSP90α and the risk of disease progression and death.

Cox proportional hazards regression model was used for univariate and multivariate analyses to validate the independent predictive role of plasmatic levels of HSP90α at diagnosis in PFS and OS. In multivariate analysis, we had adjusted the potential confounding covariates. The criterion for selecting covariates used for adjustment was that if the change in the effect estimate was more than 10% after an adjusted covariate or P value in the univariate analysis was less than 0.1, this covariate should be adjusted in Cox proportional hazards models.

All data analyses were undertaken using the statistical packages R (The R Foundation: http://www.r-project.org; version 3.4.3) and EmpowerStats software (http://www.empowerstats.com, X&Y Solutions, Inc.). A two-tailed P < 0.05 was considered statistically significant in all analyses.

## Results

### Baseline Characteristics of Selected Participants

Baseline clinical characteristics of patients disaggregated by pretreatment plasma HSP90α are listed in **Table 1**. A total of 137 patients were included in this study; the average patient age was 58.6 ± 9.8 years, and 73.7% were male. There were no significant differences in age, sex, smoking history, and ECOG-PS between the patients in the HSP90α <52.7 ng/ml group and those in the HSP90α ≥52.7 ng/ml group (all P > 0.05). However, there are more adenocarcinoma, extrathoracic metastasis, stage IV, and combination chemotherapy with pemetrexed patients in the HSP90α ≥52.7 ng/ml group (all P < 0.05).

**Table 1:** Baseline clinicopathological features.

	Total n = 137	HSP90α <52.7 ng/ml n = 58	HSP90α ≥52.7 ng/ml n = 79	P value
Age	58.6 ± 9.8			0.767
<65	94 (68.6%)	39 (67.2%)	55 (69.6%)	
≥65	43 (31.4%)	19 (32.8%)	24 (30.4%)	
Sex				0.140
Male	101 (73.7%)	39 (67.2%)	62 (78.5%)	
Female	36 (26.3%)	19 (32.8%)	17 (21.5%)	
Smoking History				0.479
Never	52 (38.0%)	24 (41.4%)	28 (35.4%)	
Ever	85 (62.0%)	34 (58.6%)	51 (64.6%)	
ECOG-PS				0.193
<2	131 (95.6%)	57 (98.3%)	74 (93.7%)	
≥2	6 (4.4%)	1 (1.7%)	5 (6.3%)	
Pathological type				0.019
Adenocarcinoma	75 (54.8%)	35 (60.3%)	40 (50.6%)	
Squamous cell carcinoma	48 (35.0%)	22 (37.9%)	26 (32.9%)	
Others	14 (10.2%)	1 (1.7%)	13 (16.5%)	
Metastasis				0.015
Intrathoracic*	78 (56.9%)	40 (69.0%)	38 (48.1%)	
Extrathoracic^	59 (43.1%)	18 (31.0%)	41 (51.9%)	
Stage				0.044
IV	106 (77.4%)	40 (69.0%)	66 (83.5%)	
IIIB/IIIC	31 (22.6%)	18 (31.0%)	13 (16.5%)	
Combination chemotherapy				0.016
Pemetrexed	33 (56.9%)	33 (41.8%)	66 (48.2%)	
Taxols^#^	25 (43.1%)	37 (46.8%)	62 (45.3%)	
Etoposide	0 (0.0%)	9 (11.4%)	9 (6.6%)	

ECOG-PS, Eastern Cooperative Oncology Group Performance Status; HSP90α, heat shock protein 90 alpha.

intrathoracic*, including lymph node metastasis (N2+N3), malignant pleural effusion, pericardial effusion, pleural metastasis, pulmonary metastasis; extrathoracic^, including bone, brain, liver, adrenal gland, and other distant metastases; Taxols^#^, including paclitaxel, paclitaxel liposome, albumin paclitaxel, and docetaxel.

### Curve Fitting of Plasmatic Levels of HSP90α and Risk of Disease Progression and Death

**Figure 2:**
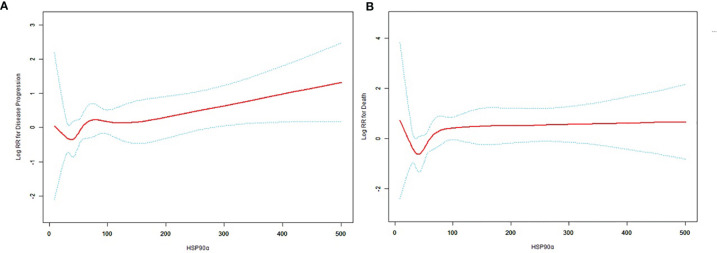
The curve fitting plot between plasmatic levels of HSP90α at diagnosis and the risk of disease progression **(A)** and death **(B)**. The estimated values and corresponding 95% confidence intervals were represented by the solid line and dashed line. The inflection point of the curve was 52.7 ng/ml.

A nonlinear relationship between plasmatic levels of HSP90α and the risk of disease progression and death (**Figure 2**) after adjusting for age, sex, smoking history, ECOG-PS, pathological type, metastasis, stage, and combination chemotherapy was shown by the smooth curve and result of Cox proportional hazards regression model with cubic spline functions. The inflection (cutoff) point was determined to be 52.7 ng/ml, which was calculated by a two-piecewise Cox proportional hazards model and recursive algorithm.

### Univariate Analysis of the Relationship Between Plasmatic Levels of HSP90α and Disease Progression and Death Risk

**Tables 2**, **3** showed the univariate analysis results. The univariate Cox proportional hazards model showed that all P values of age, sex, smoking history, ECOG-PS, pathological type, metastasis, stage, and combination chemotherapy groups were not statistically significant in predicting PFS and OS. Gratifyingly, we found that hazard ratio (HR) of disease progression was increased by 80% in the high (HSP90α ≥52.7 ng/ml) group compared to that of the low (HSP90α <52.7 ng/ml) group with 95% CI = 1.0–3.2, P = 0.036. Meanwhile, the HR of death was increased by 150% in the high (HSP90α ≥52.7 ng/ml) group compared to that of the low (HSP90α <52.7 ng/ml) group with 95% CI = 1.2–5.2, P = 0.017. These results suggested that plasmatic levels of HSP90α at diagnosis is a favorable predictor for PFS and OS in advanced lung cancer patients treated with PD-1/PD-L1 inhibitors plus chemotherapy.

**Table 2:** Univariate Cox regression analysis of the possible predictive factors for PFS.

	N	HR	P value	(95% CI)
Age				
<65	94 (68.6%)	1.0		
≥65	43 (31.4%)	1.4	0.212	(0.8, 2.4)
Sex				
Male	101 (73.7%)	1.0		
Female	36 (26.3%)	0.9	0.606	(0.5, 1.6)
Smoking history				
Never	52 (38.0%)	1.0		
Ever	85 (62.0%)	1.1	0.698	(0.7, 1.9)
ECOG-PS				
<2	131 (95.6%)	1.0		
≥2	6 (4.4%)	0.4	0.381	(0.1, 3.0)
Pathological type				
Adenocarcinoma	75 (54.8%)	1.0		
Squamous cell carcinoma	48 (35.0%)	1.0	0.969	(0.6, 1.8)
Others	14 (10.2%)	1.1	0.794	(0.5, 2.7)
Metastasis				
Intrathoracic*	78 (56.9%)	1.0		
Extrathoracic^	59 (43.1%)	1.7	0.054	(1.0, 2.8)
Stage				
IV	106 (77.4%)	1.0		
IIIB/IIIC	31 (22.6%)	0.7	0.247	(0.4, 1.3)
Combination Chemotherapy				
Pemetrexed	66(48.2%)	1.0		
Taxols^#^	62(45.3%)	1.0	0.963	(0.6,1.7)
Etoposide	9(6.6%)	1.5	0.407	(0.6,3.9)
HSP90α				
<52.7	58 (42.3%)	1.0		
≥52.7	79 (57.7%)	1.8	0.036	(1.0, 3.2)

HR, hazard ratio; CI, confidence interval; ECOG-PS, Eastern Cooperative Oncology Group Performance Status; HSP90α, heat shock protein 90 alpha; PFS, progression-free survival.

intrathoracic*, including lymph node metastasis (N2+N3), malignant pleural effusion, pericardial effusion, pleural metastasis, pulmonary metastasis; extrathoracic^, including bone, brain, liver, adrenal gland, and other distant metastases; Taxols^#^, including paclitaxel, paclitaxel liposome, albumin paclitaxel, and docetaxel.

**Table 3:** Univariate Cox regression analysis of the possible predictive factors for OS.

	N	HR	P value	(95% CI)
Age				
<65	94 (68.6%)	1.0		
≥65	43 (31.4%)	1.0	0.890	(0.5, 1.9)
Sex				
Male	101 (73.7%)	1.0		
Female	36 (26.3%)	0.5	0.110	(0.2, 1.2)
Smoking History				
Never	52 (38.0%)	1.0		
Ever	85 (62.0%)	1.5	0.235	(0.8, 3.0)
ECOG-PS				
<2	131 (95.6%)	1.0		
≥2	6 (4.4%)	0.0	0.997	(0.0, inf)
Pathological type				
Adenocarcinoma	75 (54.8%)	1.0		
Squamous cell carcinoma	48 (35.0%)	1.0	0.918	(0.5, 1.9)
Others	14 (10.2%)	1.7	0.302	(0.6, 4.5)
Metastasis				
Intrathoracic*	78 (56.9%)	1.0		
Extrathoracic ^	59 (43.1%)	1.5	0.189	(0.8, 2.8)
Stage				
IV	106 (77.4%)	1.0		
IIIB/IIIC	31 (22.6%)	0.7	0.318	(0.3, 1.5)
Combination chemotherapy				
Pemetrexed	66 (48.2%)	1.0		
Taxols^#^	62 (45.3%)	1.0	0.942	(0.5,2.0)
Etoposide	9 (6.6%)	2.5	0.099	(0.8,7.5)
HSP90α				
<52.7	58 (42.3%)	1.0		
≥52.7	79 (57.7%)	2.5	0.017	(1.2, 5.2)

HR, hazard ratio; CI, confidence interval; ECOG-PS, Eastern Cooperative Oncology Group Performance Status; HSP90α, heat shock protein 90 alpha; OS, overall survival.

intrathoracic*, including lymph node metastasis (N2 + N3), malignant pleural effusion, pericardial effusion, pleural metastasis, pulmonary metastasis; extrathoracic^, including bone, brain, liver, adrenal gland, and other distant metastases; inf, infinity, sample size was too small to calculate; Taxols^#^, including paclitaxel, paclitaxel liposome, albumin paclitaxel, and docetaxel.

### Multivariate Cox Regression Analysis

According to the results of univariate analyses, plasmatic levels of Hsp90α at diagnosis is a favorable predictor for PFS and OS in advanced lung cancer patients treated with PD-1/PD-L1 inhibitors plus chemotherapy. To validate the independent predictive role of plasmatic levels of HSP90α in PFS and OS, multivariate Cox proportional hazards models were performed (**Tables 4** and **5**). After adjusting for potential confounders (age, smoking history, ECOG-PS, and metastasis), fully adjusted models showed that when compared with the low HSP90α group, the high HSP90α group was associated with increased risk of disease progression (HR = 1.8, 95% CI = 1.0–3.2) and death (HR = 2.4, 95% CI = 1.1–5.1), all comparisons were statistically significant with P < 0.05.

**Table 4:** Unadjusted and adjusted Cox proportional hazards model for PFS.

	N	Unadjusted HR (95% CI)	P value	Fully adjusted HR (95% CI)	P value
HSP90α					
<52.7	58 (42.3%)	1.0		1.0	
≥52.7	79 (57.7%)	1.8 (1.0, 3.2)	0.036	1.8 (1.0, 3.2)	0.049

Fully adjusted model adjusts for age, smoking history, ECOG-PS, and metastasis.

HR, hazard ratio; CI, confidence interval; ECOG-PS, Eastern Cooperative Oncology Group Performance Status; HSP90α, heat shock protein 90 alpha; PFS, progression-free survival.

**Table 5:** Unadjusted and adjusted Cox proportional hazards model for OS.

	N	Unadjusted HR (95% CI)	P value	Fully adjusted HR (95% CI)	P value
HSP90α					
<52.7	58 (42.3%)	1.0		1.0	
≥52.7	79 (57.7%)	2.5 (1.2, 5.2)	0.017	2.4 (1.1, 5.1)	0.023

Fully adjusted model adjusts for age, smoking history, ECOG-PS, and metastasis.

HR, hazard ratio; CI, confidence interval; ECOG-PS, Eastern Cooperative Oncology Group Performance Status; HSP90α, heat shock protein 90 alpha; PFS, progression-free survival.

### Kaplan–Meier Survival Analysis

**Figure 3:**
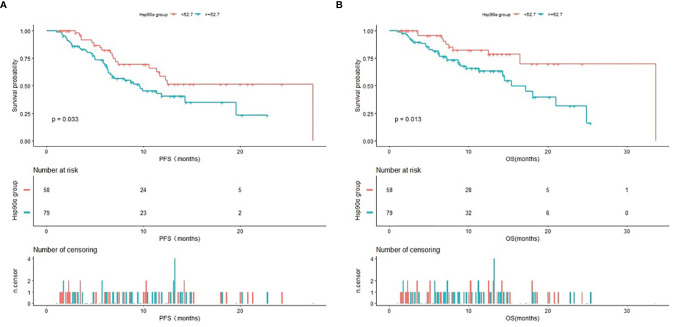
Kaplan–Meier (KM) curves of PFS **(A)** and OS **(B)** in advanced lung cancer patients treated with PD1/PD-L1 inhibitors plus chemotherapy stratified by HSP90α groups.

The Kaplan–Meier curves of PFS and OS in advanced lung cancer patients treated with PD-1/PD-L1 inhibitors plus chemotherapy stratified by HSP90α groups were shown in **Figure 3**. The Kaplan–Meier analysis showed a median PFS of 27.5 months for HSP90α <52.7 ng/ml group and 9.6 months for HSP90α ≥52.7 ng/ml group (log-rank P = 0.033); the median OS was 33.6 months with HSP90α <52.7 ng/ml group vs. 15.4 months with HSP90α ≥52.7 ng/ml group (log-rank P = 0.013).

### Stratified Analyses Using Potential Confounders as the Stratification Variables

**Figure 4:**
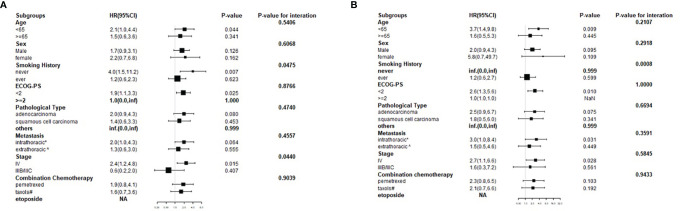
Forest plot for presenting the hazard ratio of PFS **(A)** and OS **(B)** in high HSP90α group in advanced lung cancer patients treated with PD-1/PD-L1 inhibitors plus chemotherapy.

Stratified analyses were performed to observe the trends of effect sizes on subgroups defined by covariables, including age, sex, smoking history, ECOG-PS, pathological type, metastasis, stage, and combination chemotherapy. The results of stratified and interaction analyses of the association between the high HSP90α group and the risk of disease progression and death are presented in **Figure 4**. In the high HSP90α group, patients who had no history of smoking had a higher risk of disease progression than those who had a history of smoking (HR  =  4.0 vs. HR  =  1.2, P for interaction  =  0.0475). Additionally, we observed that patients diagnosed in stage IIIB/IIIC in the high HSP90α group had a 40% lower risk for disease progression than those in the low HSP90α group. However, among patients who were diagnosed to be in stage IV, those in the high HSP90α group had a 140% higher risk for disease progression than those in the low HSP90α group; the P value for interaction analysis was 0.044, which means that the patients with high HSP90α had a significantly different HR for disease progression in different stages.

### Confirmed Objective Response Rate

When we evaluated the efficacy in the two groups of patients, the confirmed objective response rates in the two groups were similar (**Table 6**). We observed an objective response rate (ORR) of 50% in the low group and 47% in the high group. The disease control rate (DCR) was the same in both groups, and the value was 98%. However, none of the patients in the two groups could have a complete response.

**Table 6:** Summary of confirmed response assessed by RECIST version 1.1.

Confirmed Response	HSP90α <52.7 ng/ml n = 58	HSP90α ≥52.7 ng/ml n = 79
**Best response**		
**Complete response (CR)**	0 (0%)	0 (0%)
**Partial response (PR)**	29 (50%)	37 (47%)
**Stable disease (SD)**	28 (48%)	40 (51%)
**Progressive disease (PD)**	0 (0%)	2 (2%)
**Not evaluable**	1 (2%)	0 (0%)
**Objective response rate(ORR)**	50%	47%
**Disease control rate (DCR)**	98%	98%

Objective response rate(ORR) = Complete response (CR) + Partial response (PR).

Disease control rate (DCR) = Complete response (CR) + Partial response (PR) + Stable disease (SD).

Not evaluable = Patients who did not have one postbaseline imaging assessment.

RECIST, Response Evaluation Criteria In Solid Tumors; HSP90α, heat shock protein 90 alpha.

## Discussion

As we all know, tumor progression not only depends on the tumor’s oncogene or tumor suppressor gene but also on host-related factors. HSPs are essential molecular chaperones in humans, which contribute to tumorigenesis, tumor cell viability, and tumor progression by regulating the biological function of several protein kinases, oncogenes, protein phosphatases, transcription factors, and cofactors (13–15). HSP90, an essential member of the HSP family, has two subtypes, including HSP90α and HSP90β. Under normal conditions, HSP90α and HSP90β are present in cells, with deficient levels in blood circulation; in a condition of hypoxia, injury, oxidation, and other stresses, HSP90α can be secreted explicitly to the extracellular space (16, 17). It has been demonstrated that plasma levels of HSP90α were higher in most patients with cancer, and the expression of HSP90α may be prognostic markers in several solid tumors, including lung cancer, liver cancer, and breast cancer (18–20). Thus, HSP90α is emerging as a hot topic in the research of cancer.

In the present study, we identified that high HSP90α was associated with poor PFS and OS in advanced lung cancer patients treated with PD-1/PD-L1 inhibitors plus chemotherapy after adjusting other covariates, suggesting that plasmatic levels of HSP90α at diagnosis may serve as a promising prognostic indicator in clinical practice. Meanwhile, we found that the patients with high HSP90α had a significantly different HR for disease progression in different stages.

We reviewed the literature in the Pubmed database and found that previous studies reported that HSP90α might be a prognostic marker of lung cancer. In the study by Shi et al. (9), plasma HSP90α protein levels could predict the responses of patients with lung cancer to chemotherapy. The research of Zhong et al. (8) indicated that plasma HSP90α was considered a valuable predictor of early chemotherapy effectiveness in advanced non-small cell lung cancer. In the study by Du et al. (21), HSP90α attenuates the efficacy of anticancer drugs in small cell lung cancer. However, no previous studies have focused explicitly on the relationship between plasmatic levels of HSP90α at diagnosis and the prognosis of advanced lung cancer patients treated with PD1/PD-L1 inhibitors plus chemotherapy. This is the first report studied on the predictive significance of plasmatic levels of HSP90α at diagnosis in advanced lung cancer patients treated with PD1/PD-L1 inhibitors plus chemotherapy.

Although the mechanism by which high HSP90α was associated with poor PFS and OS in advanced lung cancer patients treated with PD-1/PD-L1 inhibitors plus chemotherapy remains unknown, there were some plausible explanations. The HSP90α on the surface of autophagosomes Tumor-Released Autophagosomes (TRAPs) released by tumor cells stimulates CD4+ T cells to produce Interleukin-6 (IL-6) through the Toll-like receptors (TLR2)–myeloid differentiation factor88 (MyD88)–nuclear factor-k-gene binding (NF-κB) signal cascade, and the autocrine IL-6 induced by TRAPs further promotes the secretion of IL-10 from CD4+ T cells. And IL-21 CD4+ T cells triggered by STAT3 and TRAPs inhibit the function of CD4+ and CD8+ effector T cells in an IL-6- and IL-10-dependent manner, which affected the efficacy of immunotherapy (22). HIF-1α regulates the secretion of HSP90α (23), while hypoxia inducible factor-1 (HIF-1α) is a significant regulator of PD-L1 mRNA and protein expression in lung cancer (24, 25). Thus, we could speculate that the higher expression of plasma HSP90α, the more lung cancer tissues with HIF-1α positivity, which may affect the expression of PD-L1, affecting the efficacy of immunotherapy. A recent study showed that the efficacy of immunotherapy in lung cancer patients is closely related to the expression of HLA-A*01 and/or A*02 alleles (26). At the same time, a study had shown that HSP90 was related to the regulation of human leukocyte antigen (HLA) expression (27); this also gives a new direction to elucidate further the relationship between HSP90 and the efficacy of immunotherapy. Further research is required to elucidate the underlying mechanisms.

Generally, the optimal cutoff values can be determined by three methods: biostatistical software, cubic spline functions, and smooth curve fitting combined with flection-point calculation and receiver operating characteristic (ROC) curve. In our study, the optimal cutoff value of plasmatic levels of HSP90α at diagnosis was determined by cubic spline functions and smooth curve fitting combined with flection-point calculation. However, there is no consensus on the appropriate cutoff value of plasmatic levels of HSP90α at diagnosis for predicting prognosis. For example, Shi et al. (9) used a cutoff point of 56.33 ng/ml to predict the responses of patients with lung cancer to chemotherapy, which is close to the cutoff value in our study. On this basis, we are reasonably confident that the cutoff value of plasmatic levels of HSP90α at diagnosis in our study is reliable.

Our study had several strengths. First, this is the first study to evaluate the predictive value of plasmatic levels of HSP90α at diagnosis in advanced lung cancer patients treated with PD-1/PD-L1 inhibitors plus chemotherapy. Secondly, we used strict statistical adjustment to minimize residual confounders to elucidate the relationship between plasmatic levels of HSP90α and PFS and OS. Last but not least, we conducted a subgroup analysis to verify our robust and stable results in specific subgroups. Although these results are not enough to directly influence clinical practice at present, they would be helpful for future research on the establishment of diagnostic or predictive models of PFS and OS for advanced lung cancer patients treated with PD-1/PD-L1 inhibitors plus chemotherapy. They could not only serve as clinical monitoring indexes in clinical settings that may aid appropriate risk stratification and treatment decision-making but also further formulate individualized treatment schemes. A closer follow-up should be conducted once plasmatic levels of HSP90α ≥52.7 ng/ml are identified at diagnosis.

Despite our important findings, there were some limitations to the present study. First, the cases were collected from a single institute; because of the retrospective design of our study, it is easy to introduce selection bias and may distort the observed association. Second, we focused on the plasmatic levels of HSP90α at diagnosis but failed to analyze the dynamic changes in the HSP90α value during the whole process. Third, we excluded the patients who had an active infection or inflammatory diseases before blood examination, as they may influence the value of HSP90α, so that the conclusion of our study cannot be applied to these patients. Therefore, further large-scale prospective clinical trials and basic research studies are required to elucidate the relationship between plasmatic levels of HSP90α at diagnosis and poor prognosis in advanced lung cancer patients treated with PD1/PD-L1 inhibitors plus chemotherapy.

## Conclusion

In conclusion, plasmatic levels of HSP90α at diagnosis can be considered a potential independent prognostic marker of advanced lung cancer patients treated with PD-1/PD-L1 inhibitors plus chemotherapy. A further large-scale prospective validation study is needed to determine whether these results are widely applicable.

## Data Availability Statement

The raw data supporting the conclusions of this article will be made available by the authors without undue reservation.

## Ethics Statement

The studies involving human participants were reviewed and approved by the ethics committee of Guangxi Medical University Cancer Hospital. The patients/participants provided their written informed consent to participate in this study.

## Author Contributions

QY contributed to the conception of the study and performed the research. SC performed the data analyses and wrote the article. SZ helped to perform the analysis with constructive discussions and make revisions to the paper. All authors contributed to the article and approved the submitted version.

## Conflict of Interest

The authors declare that the research was conducted in the absence of any commercial or financial relationships that could be construed as a potential conflict of interest.

## Publisher’s Note

All claims expressed in this article are solely those of the authors and do not necessarily represent those of their affiliated organizations, or those of the publisher, the editors and the reviewers. Any product that may be evaluated in this article, or claim that may be made by its manufacturer, is not guaranteed or endorsed by the publisher.
